# Acoustic Features Influence Musical Choices Across Multiple Genres

**DOI:** 10.3389/fpsyg.2017.00931

**Published:** 2017-07-04

**Authors:** Michael D. Barone, Jotthi Bansal, Matthew H. Woolhouse

**Affiliations:** ^1^Psychology, Neuroscience and Behaviour, McMaster UniversityHamilton, ON, Canada; ^2^School of the Arts, McMaster UniversityHamilton, ON, Canada

**Keywords:** Nokia DB, acoustic features, musical preference, musical genre, music downloads, musical influence, music information retrieval

## Abstract

Based on a large behavioral dataset of music downloads, two analyses investigate whether the acoustic features of listeners' preferred musical genres influence their choice of tracks within non-preferred, secondary musical styles. Analysis 1 identifies feature distributions for pairs of genre-defined subgroups that are distinct. Using correlation analysis, these distributions are used to test the degree of similarity between subgroups' main genres and the other music within their download collections. Analysis 2 explores the issue of main-to-secondary genre influence through the production of 10 feature-influence matrices, one per acoustic feature, in which cell values indicate the percentage change in features for genres and subgroups compared to overall population averages. In total, 10 acoustic features and 10 genre-defined subgroups are explored within the two analyses. Results strongly indicate that the acoustic features of people's main genres influence the tracks they download within non-preferred, secondary musical styles. The nature of this influence and its possible actuating mechanisms are discussed with respect to research on musical preference, personality, and statistical learning.

## 1. Introduction

This paper concerns the degree to which the acoustic features of a person's preferred musical genre influence their choice of songs or tracks within non-preferred, secondary musical styles. For example, do people who favor Dance music, which is typically faster in tempo than other styles, listen to up-tempo Jazz and/or Reggae tracks, rather than slower examples of these genres? Similarly, might someone whose preference is for Metal also gravitate toward relatively dynamic, “high-octane” Country or Blues music (assuming, of course, that those genres are not mutually exclusive; Bansal and Woolhouse, 2015). Although conceptually straightforward, this question addresses active research areas within the fields of music cognition and Music Information Retrieval (MIR), and, to some extent, highlights current limitations within both. Firstly, the phenomenon of features within a preferred genre influencing song selection within secondary musical styles falls under the general topic of “cognitive leakiness,” a notion explored in depth in the area of consumer choice and commerce (e.g., Rieskamp et al., 2006), but less so in music perception. Secondly, topics involving musical features, in this case extracted from audio, by necessity utilize MIR techniques (e.g., Lartillot and Toiviainen, 2007). The psychological reality of extracted acoustic features is an open question (Friberg and Schoonderwaldt, 2014), and therefore research demonstrating their influence upon musical preference, may, in part, help to legitimize their perceptual existence.

In attempting to investigate song selection and acoustic-feature influence, our study brought music cognition and MIR together within the context of “big data” (Russom, 2011). The data in question consisted of ca 1.3 billion music downloads made by approximately 17 million users in multiple countries between 2007 and 2014. Somewhat anticipating our results, the following analyses demonstrate significant effects with respect to 10 extracted acoustic features, and 10 subgroups of users defined by preferred musical genre. Before describing the data, methodology, and reporting our results, we first review literature that addresses factors responsible for, and that influence, device usage and decision making, including song selection.

Similarly to the devices used in our study, which were mobile phones (see Section 2), Butt and Phillips (2008) sought to predict amounts and types of mobile-phone use from 120 participants rated for extraversion, agreeableness, conscientiousness, neuroticism, and self-esteem (using the Coopersmith self-esteem inventory; Coopersmith, 1959). Individuals assessed as being neurotic, disagreeable, unconscientious, and/or extraverted tended to spend more time messaging using SMS; disagreeable extraverts changed cellphone backgrounds and ringtones more frequently, indicating phone use as a means of stimulation and/or diversion; individuals who scored highly in neuroticism had relatively greater internet use, according to the authors, perhaps in an attempt to overcome loneliness. In sum, Butt and Phillips (2008) concluded that psychological profiling with respect to established personality dimensions could robustly explain how people chose to use their mobile phones.

While successfully modeling human behavior, some researchers (e.g., Ross et al., 2009) have argued that the personality traits referred to above may be too general to model online behavior, including cellphone usage. For example, Hughes et al. (2012) investigated whether a lower-order, relatively narrow personality facet such as Need for Cognition (NFC) was able to predict online social and information-seeking behaviors. NFC is an individual's predisposition to engage with and enjoy information and cognitive endeavors, e.g., news content, crossword puzzles, Sudoku (Haugtvedt et al., 1992; Verplanken, 1993). Despite its specificity, as opposed to a broader dimension such as openness, NFC in the study conducted by Hughes et al. (2012) was found to correlate positively with Twitter usage, presumably due to this social-networking service's relatively high information content. Those with high ratings for sociability and extraversion appeared to prefer Facebook. For additional research concerning social media and personality, see Moore and McElroy (2012).

In addition to device- and personality-specific research, studies exploring the interconnectedness of various forms of media and the consumption of culture, including music, have been undertaken. Finn (1997), in a diary study of over 200 university students, correlated radio listening, TV watching, pleasure reading, and moviegoing with openness, conscientiousness, extraversion, agreeableness, and neuroticism (referred to as the Big Five personality traits; see Costa and MacCrae, 1992). The strongest relationship for mass-media use was between openness and pleasure reading; extraversion was negatively associated with pleasure reading, as was openness and watching TV. Rentfrow and Gosling (2003) assert that the perception of a musical genre depends in part upon the social setting in which it is heard and, by extension, the medium through which it is accessed; in other words, that people's preferences for certain media over others may influence musical categorization. With respect to music listening, where radio continues to play a major role (Peoples, 2015), personality studies have uncovered multiple associations: openness with Blues and Jazz; conscientiousness with Soul and Funk; extraversion with Pop and Rap, and so on (Zweigenhaft, 2008; see also Rentfrow and Gosling, 2003). Moreover, personality appears not only to influence the extent to which individual genres are chosen, but also the overall heterogeneity of our musical tastes, i.e., whether we possess narrow or wide-ranging music-listening habits (Rawlings and Ciancarelli, 1997). In sum, personality research provides evidence for the existence of an overarching psychological framework in which effects akin to cognitive leakiness may occur (Rieskamp et al., 2006). As the research outlined above indicates, personality is a potent phenomenon, suffusing, guiding, and shaping our decisions, including the seemingly inconsequential behavior of choosing music.

In contrast to personality, which is assumed to be relatively stable over time (Leon et al., 1979), mood can undergo rapid affective swings (McFarlane et al., 1988). Moreover, while research has tended to concentrate on how music influences or induces mood, particularly with respect to consumer choice (e.g., Kim and Areni, 1993; North et al., 1999), the converse is also true: mood influences musical choice (Friedman et al., 2012). Which is to say, assuming environmental factors and personal histories to be equal, a person's musical preferences do not depend solely on their personality, but, in addition, are subject to spur-of-the-moment choices influenced by mood.

Amongst the theoretical models advanced to elucidate the role of mood in decision-making, perhaps the most influential is the Affect Infusion Model (AIM), developed by Joseph Forgas in the early 1990s (Forgas, 1995). In brief, the AIM seeks to explain how mood determines a person's capacity to process information—the importance of mood tends to increase in situations involving heavy cognitive load. As information complexity rises, and redundancy falls, the influence of mood on an individual's evaluations and responses increases, resulting in “intuitive” decision-making. Presumably, therefore, when faced with a plethora of diverse musical artists, tracks, and genres, people tend to rely more upon their current mood, in which case the influence of personality may be temporarily reduced or suspended. To the authors' knowledge, within the domain of music-preference research, this hypothesis has yet to be tested.

Despite this possible lacuna within the experimental literature, paradigms employing music-induced moods have produced results that are consistent with aspects of the AIM model. For example, risk-taking varies when mood is induced through listening to preferred vs. disliked music. In a real-money gambling study, in which participants placed bets during either music-liked and disliked trial blocks, Halko and Kaustia (2015) found that people's appetites for risk-taking significantly increased when listening to preferred tracks. They conjectured that listening to preferred types of music increases the “marginal utility” of money (i.e., the additional satisfaction someone gains from consuming a good or service; Kauder, 2015), which, in turn, increases the likelihood of participating in gambling. Furthermore, Halko and Kaustia (2015) argued that their results are supported by recent studies in neuroscience. Berns et al. (2010) have found levels of activation in reward areas of the brain (e.g., nucleus accumbens) to be proportional to the degree to which music is liked. The behavioral effect of music on risk-taking also co-varies with brain activation in the amygdala and the dorsal striatum (Halko et al., 2015), key brain regions associated with the calculation and assessment of value. In short, in addition to its mood-inducing properties, music listening gives rise to distributed neurological operations in which functionally differentiated networks are simultaneously activated. For a review of research relating to the induction of mood through music, see Västfjäll (2002).

While mood and personality pertain, to some degree, to an individual, shared demographic factors, including culture, education, sex, and age appear to affect people's musical choices (Christenson and Peterson, 1988; Roberts and Henriksen, 1990; Peterson and Kern, 1996; LeBlanc et al., 1999; Schäfer and Sedlmeier, 2009; North and Davidson, 2013). Of these, age is the strongest predictor of musical preference (Christenson and Peterson, 1988). Older adolescents prefer ‘lighter’ qualities in music compared to younger adolescents (Roberts and Henriksen, 1990). General enjoyment of music from Grade 1 to college drops for a time until rising around the age of puberty, following a U-shaped curve across development (LeBlanc et al., 1996). Supported by cross-cultural studies, sociological research suggests that preferences for eclectic artists rise as national education values improve (Peterson and Kern, 1996). With respect to sex, a music-choice study suggested that males prefer music with themes of dominance and independence, whereas females preferred music with relationship and emotion themes (Christenson and Peterson, 1988). However, the extent to which this research is generalizable is open to debate: almost 30 years has elapsed since Christenson and Peterson's study, which was based on low-sample surveys with relatively little demographic variation. Furthermore, LeBlanc et al. (1999) and North and Davidson (2013) found that demographic information did not conclusively determine music preferences; two- and three-way interactions were found between age, sex and country, and controlling for these factors significantly reduced the strength of the relationships.

Although the foregoing covers aspects of decision-making involving music, none of the research and experimental scenarios referred to above necessarily replicate, or are fully applicable to, the particular issue at hand; namely, the degree to which the features of a person's preferred musical genre influence their choice of tracks within non-preferred, secondary musical styles. A primary motivation for undertaking this research was because, to our knowledge, musical-feature influence has yet to be investigated using large behavioral data sets. Despite not containing user-personality information *per se*, our database of global music consumption afforded us the opportunity to undertake research in this hitherto underexplored area, and, in the process, develop a series of relatively novel analytical methods. The study is divided into two main analyses. Using correlation, Analysis 1 identified differences in feature-dispersion patterns of genre-defined subgroups of users. Analysis 2 involved the exhaustive calculation of feature-influence matrices, which, in combination with central-tendency statistics, were used to detect the influence of main-genre features on those of secondary genres. The methods and results of each analysis follow a description of the data.

## 2. Data

### 2.1. Database

This study utilized a global music-download database, consisting primarily of music metadata made by people downloading tracks and albums onto Nokia mobile phones. The data became accessible through a data-sharing agreement between McMaster University and the Nokia Corporation, begun in 2012; the aim of the agreement was to facilitate sociocultural and musicological research relating to global music consumption. In January 2015, the Nokia division responsible for online music became a separate entity under the name MixRadio; MixRadio ceased commercial operations in February 2016. Henceforth, we refer to the data as pertaining to the Nokia DB^1^.

Nokia DB contains downloads from 32 countries, representing every major continent, made between November 2007 and September 2014. In total there are over 1.36 billion track-downloads, relating to a subset of ca 17 million user accounts, and approximately 36 million tracks, written and/or performed by over one million artists. Following the purchase of a mobile device, users could explore artists and tracks without further cost constraints via online stores. Each download's metadata includes information such as track name, artist, album, anonymous user identifier (ID), date, local time, country, and artist-level genre. Supplied by record labels, in total there are 63 genre tags, ranging from mainstream (Country, Pop, Rock) to relatively obscure (Ambient, Flamenco, Khaleeji). For additional information and research concerning Nokia DB, see Woolhouse and Bansal (2013), Woolhouse et al. (2014), Woolhouse and Renwick (2016).

### 2.2. Data enrichment

Prior to embarking upon this study, we enriched the download metadata with acoustic features from open-source databases, including The Echo Nest (Bertin-Mahieux et al., 2011). As of May 2016, The Echo Nest application programming interface (API) was subsumed by Spotify; henceforth, for the sake of simplicity, we refer to all extracted acoustic features in our analyses as relating to Spotify. Examples of acoustic features accessed from the Spotify Web API^2^ include Acousticness, Danceability, Duration, Energy, Instrumentalness, Liveness, Loudness, Speechiness, Tempo, and Valence. The data are arranged in a relational database management system and queried using the open-source MySQL 5.1 implementation of SQL (Groff and Weinberg, 2002). In addition, the Python Database API (Lemburg, 2008) enabled more extensive, iterative analyses to be undertaken.

### 2.3. Acoustic features

Of the 36 million songs available in Nokia DB, 9 million have been linked to the 10 high- and low-level acoustic features (McKay, 2004, p. 10) listed below. A brief description of each feature now follows; see Jehan and DesRoches (2011) for further information^3^.

**Acousticness.** Value representing the probability that a track was created using acoustic instruments, including voice. Float; range, 0–1.
**Danceability.** A track's “foot-tapping” quality, based on tempo, rhythm stability, beat strength, and isochrony. Float; range, 0–1.
**Duration.** The duration of a track in seconds as calculated by the Spotify analyzer. Float; maximum value, 6,060 s.
**Energy.** A perceptual estimation of frenetic activity throughout a track. High-Energy tracks have increased entropy, and tend to feel fast, loud, and noisy (e.g., Death Metal). Float; range, 0–1.
**Instrumentalness.** Value representing the probability that a track was created using only instrumental sounds, as opposed to speech and/or singing. Float; range, 0–1.
**Liveness.** Value representing the probability that a track was recorded in the presence of an audience rather than in a studio. Float; range, 0–1.
**Loudness.** The average loudness of a track in decibels. Loudness is the psychological correlate of signal amplitude.
**Speechiness.** Value representing the presence of spoken words in a track, e.g., talk show, audio book, poetry, rap. Float; range, 0–1.
**Tempo.** The estimated tempo of a track in beats per minute. Float; range, 0–294.
**Valence.** A perceptual estimation of a tracks positive/negative affect, e.g., happy and cheerful, or sad and depressed. Float; range, 0–1.

### 2.4. X-heads

The behavioral aspects of our analyses were based on the categorization of users into “X-head” subgroups, where X was the most numerous genre. For example, a user with a majority of Metal downloads was classified as a Metal-head; most Classical downloads, a Classical-head, and so on. This enabled us to identify groups of users that were more accustomed, so we assume, to one particular genre than another, and, thus, attuned to the acoustic features prevailing within that genre. In rare instances where no genre had an absolute majority, the genre of the chronologically earliest download determined a user's categorization.

Our intention was for the definition of an X-head to be as straightforward as possible, and hence our simple criterion of a majority of downloads of a particular genre. In order to keep our study within manageable parameters, 10 X-head subgroups were selected for investigation: Bollywood, Classical, Dance, Jazz, Mandarin Pop, Metal, Pop, Rap/Hip Hop, Reggae, and Rock. Two primary reasons determined this choice: (1) these are amongst the most heavily downloaded genres within Nokia DB; and (2) they include culturally distinct genres, some of which are perhaps less well represented in music-psychology research, e.g., Mandarin Pop. Table 1 shows the total number of users and tracks per X-head subgroup entered into the analyses.

**Table 1:** Descriptive statistics for X-head subgroups: number of users; number of downloads (average downloads per user).

**X-head subgroup**	**Users**	**Downloads**
Pop	3,215,135	74,421,204 (23.14)
Bollywood	2,134,919	30,340,368 (14.21)
Mandarin Pop	1,944,975	15,165,454 (7.80)
Rock	349,205	23,489,799 (67.27)
Dance	163,388	1,676,634 (10.26)
Rap/Hip Hop	156,384	2,122,862 (13.57)
Metal	94,015	4,513,560 (48.01)
Classical	45,903	2,260,496 (49.25)
Jazz	22,644	1,131,941 (50.00)
Reggae	13,530	240,615 (17.63)

## 3. Analysis 1: feature distributions

### 3.1. Method

**Figure 1:**
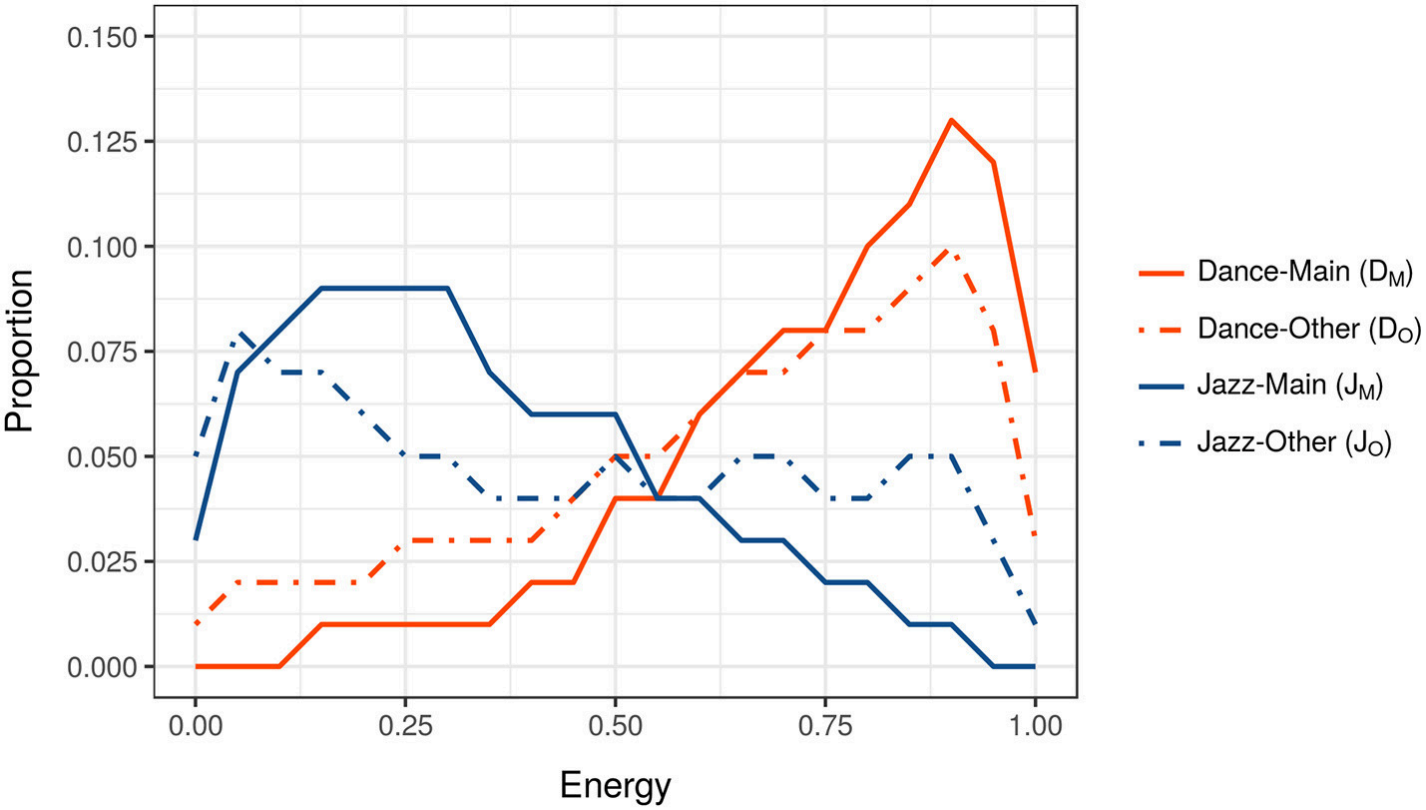
Energy distributions of tracks belonging to Dance-head and Jazz-head subgroups. The orange lines, *D*_*M*_ and *D*_*O*_, show the distributions of tracks downloaded by Dance-heads; the blue lines, *J*_*M*_ and *J*_*O*_, show the distributions of tracks downloaded by Jazz-heads.

**Figure 2:**
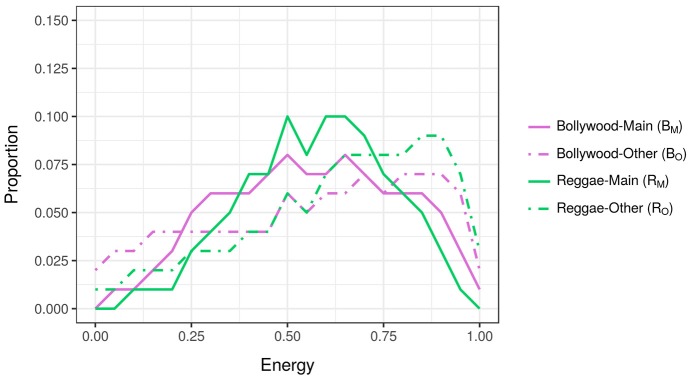
Energy distributions of tracks belonging to Bollywood-head and Reggae-head subgroups. The purple lines, *B*_*M*_ and *B*_*O*_, show the distributions of tracks downloaded by Bollywood-heads; the green lines, *R*_*M*_ and *R*_*O*_, show the distributions of tracks downloaded by Reggae-heads.

The initial task in Analysis 1 was to identify feature distributions for pairs of X-head subgroups that were distinct. The reason for this is illustrated in Figures 1, 2. Figure 1 shows the Energy distributions of tracks belonging to two X-head subgroups: the solid-orange line, *D*_*M*_, shows the distribution for Dance tracks downloaded by Dance-heads (*Dance*_*Main*_ = *D*_*M*_); the solid-blue line, *J*_*M*_, shows the distribution for Jazz tracks downloaded by Jazz-heads (*Jazz*_*Main*_ = *J*_*M*_). Notice that the peak of *D*_*M*_ is to the right, while the peak of *J*_*M*_ is to the left. The two peaks' relative positions indicate that, in general, Dance tracks listened to by Dance-heads have higher Energy than Jazz tracks listened to by Jazz-heads, as calculated by the Spotify analyzer.

Also present within Figure 1 are lines that show Energy distributions belonging to Dance- and Jazz-heads, but for tracks other than their predominant genres: the dotted-orange line, *D*_*O*_, shows the distribution of non-Dance tracks downloaded by Dance-heads (*Dance*_*Other*_ = *D*_*O*_); the dotted-blue line, *J*_*O*_, shows the distribution of non-Jazz tracks downloaded by Jazz-heads (*Jazz*_*Other*_ = *J*_*O*_). Two things are important to note: (1) the Energy distribution of Dance-heads' non-Dance tracks mirrors the distribution of their Dance tracks, e.g., both *D*_*M*_ and *D*_*O*_ peak on the right; and (2) the Energy distribution of Jazz-heads' non-Jazz tracks mirrors the distribution of their Jazz tracks, e.g., both *J*_*M*_ and *J*_*O*_ peak on the left. Which is to say, when Dance-heads download non-Dance tracks, there is a tendency for these tracks to be similar in terms of Energy to Dance tracks. Alternatively put, the generally high Energy of Dance tracks influences the choices Dance-heads make with respect to non-Dance music, while the generally low Energy of Jazz tracks influences the choices Jazz-heads make with respect to non-Jazz music.

The observation above relies upon X-head pairs having dissimilar feature distributions (i.e., lines *D*_*M*_ and *J*_*M*_), and, in the case of Figure 1, the distribution of *D*_*M*_ being closer to *D*_*O*_ than *J*_*O*_, and *J*_*M*_ being closer to *J*_*O*_ than *D*_*O*_. If, however, the distributions of the X-heads' main genres are homologous, as is the case for Bollywood- and Reggae-heads in Figure 2 (solid green and purple lines), then no such pattern of similar/different distributions is possible. Which is to say, distributions where X-heads' main genres are more-or-less similar, are less able to demonstrate acoustic-feature influence.

**Figure 3:**
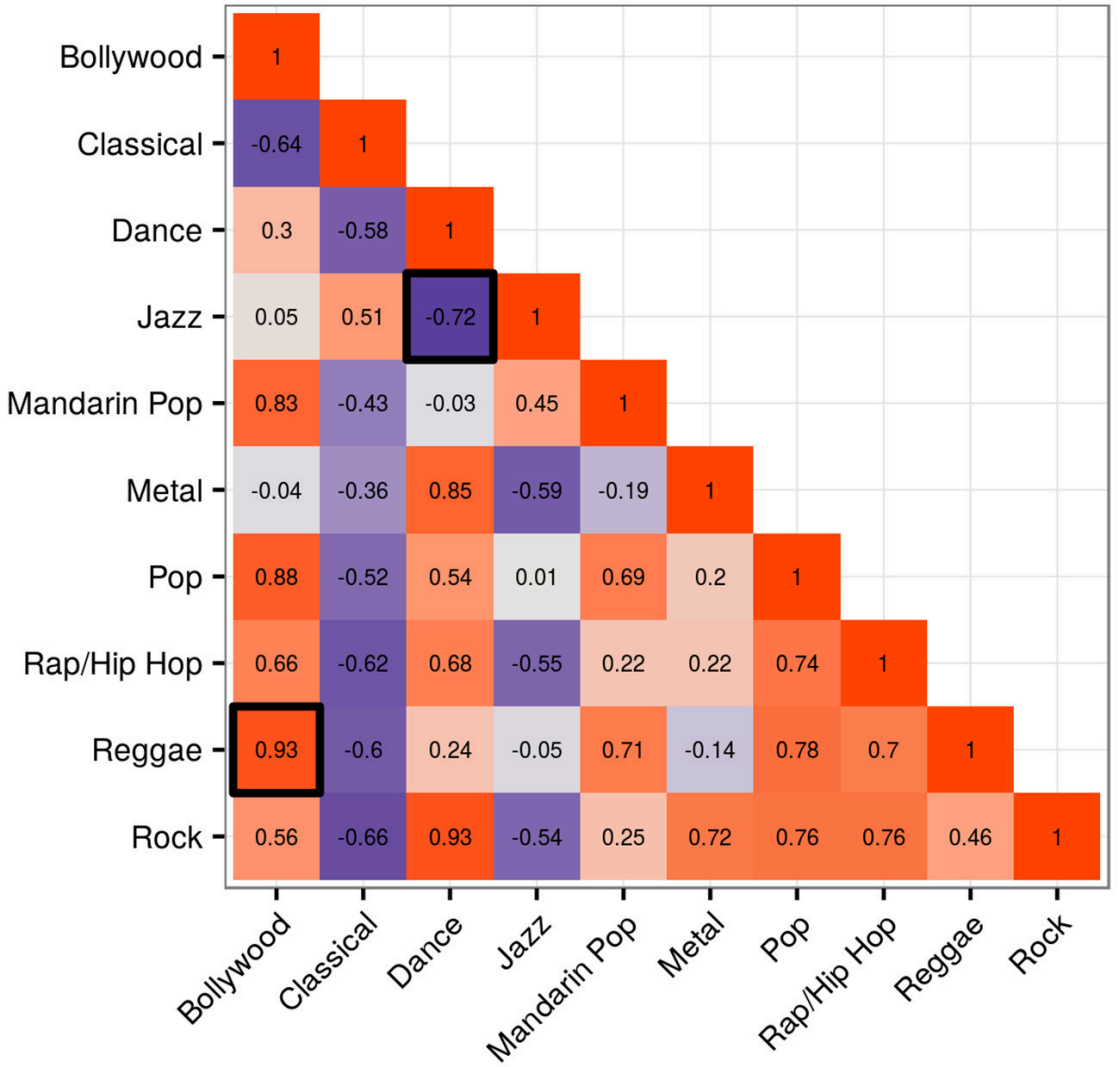
Correlation matrix for Energy showing coefficients between pairs of X-head subgroups. The two highlighted cells relate to the solid lines in Figures 1, 2, Dance and Jazz, and Bollywood and Reggae respectively.

The distributions for all possible X-heads' main genres were correlated with each other in order to identify pairs with dissimilar distributions. This was conducted for all 10 features. Using Pearson product-moment correlation, five features yielded no negative coefficients, and were thereby eliminated from the analysis. The five remaining features yielding negative coefficients, usable in the analysis, included Acousticness, Danceability, Energy, Loudness, and Valence. Figure 3 shows the correlation matrix for feature Energy. The two highlighted cells within the matrix relate to the solid lines in Figures 1, 2, Dance and Jazz, and Bollywood and Reggae respectively. The Dance-Jazz coefficient is negative (reflected in the dissimilar distributions in Figure 1); the Bollywood-Reggae coefficient is positive (reflected in the similar distributions in Figure 2). In the case of Energy, this process yielded 19 X-head pairs suitable for analysis, i.e., 19 cells with negative coefficients.

Following this, for each X-head pair AB, the distribution of A's main genre (e.g., *D*_*M*_, Figure 1) was correlated with the distribution of A's other music (e.g., *D*_*O*_). Next, the distribution of A's main genre (e.g., *D*_*M*_) was correlated with the distribution of B's other music (e.g., *J*_*O*_). This produced two coefficients. This process was then repeated for B: B's main genre (e.g., *J*_*M*_) was correlated with the distribution of their other music (e.g., *J*_*O*_), and the distribution of B's main genre (e.g., *J*_*M*_) was correlated with the distribution of A's other music (e.g., *D*_*O*_). A and B together, therefore, produced four coefficients. For each feature, this was repeated for all X-head pairs with negatively correlated distributions, and the resulting coefficients entered into a paired sample *t*-tests in which “within-group” coefficients (e.g., *D*_*M*_ correlated with *D*_*O*_) were paired with “between-group” coefficients (e.g., *D*_*M*_ correlated with *J*_*O*_).

**Figure 4:**
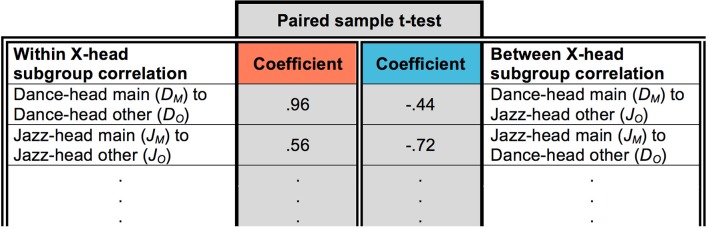
Example of paired sample *t*-test with respect to Energy in which within-group coefficients were paired with between-group coefficients. Only the first two from 38 pairs are shown.

Figure 4 illustrates this process for Energy with respect to Dance- and Jazz-heads. In total, the 19 X-head pairs identified in the Energy correlation matrix in Figure 3 gave rise to a *t*-test into which 38 pairs were entered. This enabled us to observe whether there was a closer relationship between the features of A's main genre and their other music (Figure 4, red column; e.g., *D*_*M*_ and *D*_*O*_) than with the features of B's other music (Figure 4, blue column; e.g., *D*_*M*_ and *J*_*O*_) and vice versa, i.e., whether there was a significant influence of the main genre on music of secondary importance within people's downloads. If there had been no influence, then the distributions of either A or B's other music (e.g., *D*_*O*_ or *J*_*O*_) would not be expected to show a consistently closer relationship to their respective main genre distributions (e.g., *D*_*M*_ or *J*_*M*_). The results of this analysis for the five viable features referred to above are now presented.

### 3.2. Results

**Figure 5:**
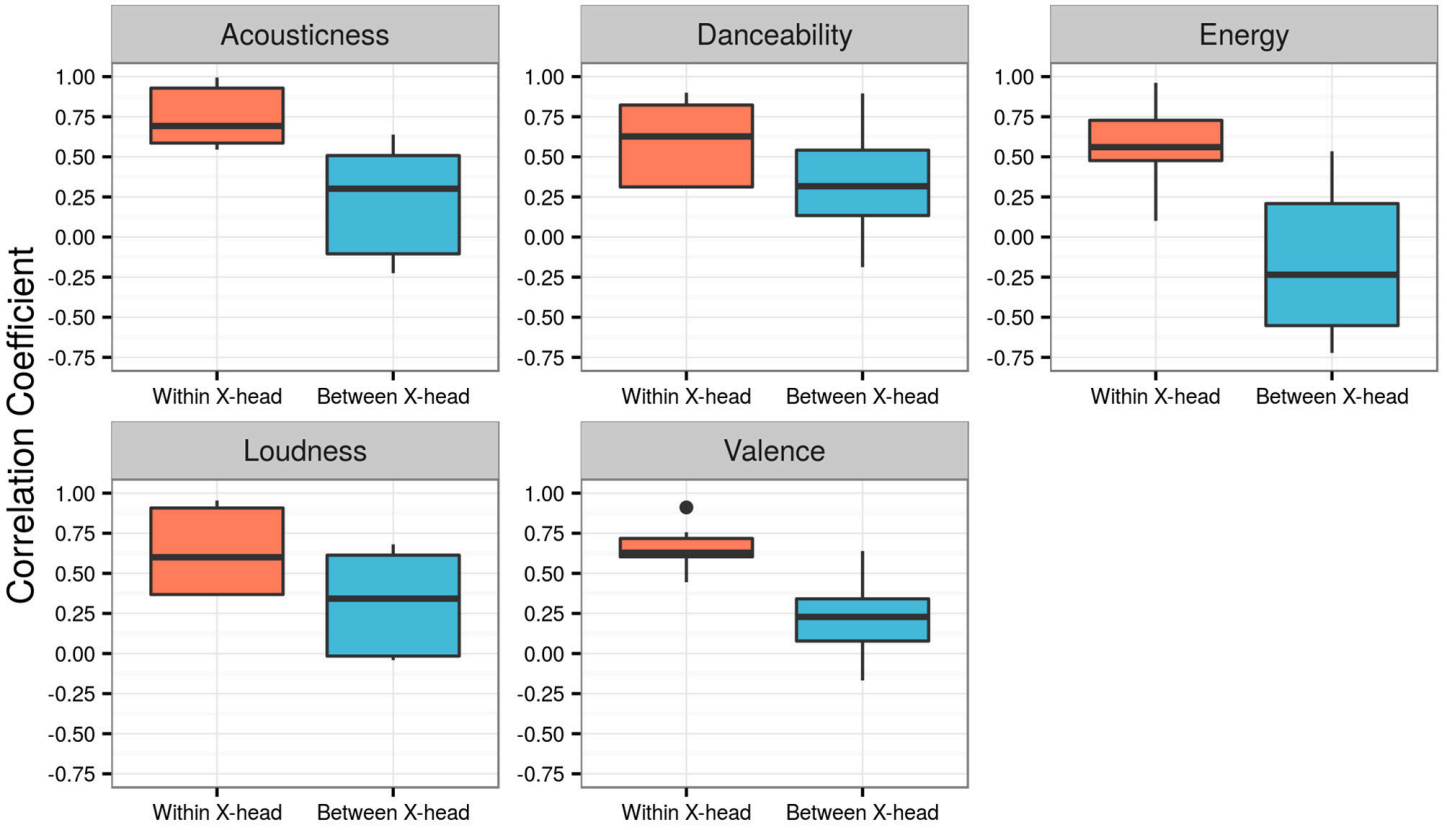
Boxplots of the five features within Analysis 1. Red boxes represent the within X-head coefficients; blue boxes represent the between X-head coefficients.

As previously described, the presence of negatively correlated distributions, shown in Figure 3 with respect to Energy, enabled the influence of five features to be studied using the present methodology. In sum, Acousticness had 12 negatively correlated distributions, Danceability 10, Energy 19, Loudness 5, and Valence 9 (see Supplementary Figure 1). Figure 5 shows boxplots of the five features within the analysis. The red boxes on the left of each graph represent the within X-head coefficients; blue boxes on the right are the between X-head coefficients. Paired-sample *t*-tests, conducted to compare the within X-head coefficients and between X-head coefficients, showed the following results (sig. 2-tailed):

**Acousticness.** Significant difference for within (*M* = 0.749, *SD* = 0.170) and between (*M* = 0.250, *SD* = 0.302) X-head coefficients; *t*(23) = 11.887, *p* < 0.0001.
**Danceabilty.** Significant difference for within (*M* = 0.608, *SD* = 0.225) and between (*M* = 0.313, *SD* = 0.288) X-head coefficients; *t*(19) = 8.046, *p* < 0.0001.
**Energy.** Significant difference for within (*M* = 0.557, *SD* = 0.233) and between (*M* = 0.174, *SD* = 0.414) X-head coefficients; *t*(37) = 9.110, *p* < 0.0001.
**Loudness.** Significant difference for within (*M* = 0.636, *SD* = 0.270) and between (*M* = 0.315, *SD* = 0.301) X-head coefficients; *t*(9) = 10.656, *p* < 0.0001.
**Valence.** Significant difference for within (*M* = 0.653, *SD* = 0.113) and between (*M* = 0.223, *SD* = 0.199) X-head coefficients; *t*(17) = 11.887, *p* < 0.0001.

### 3.3. Discussion

The statistics above confirm what is clearly evident in the boxplots in Figure 5: there is a significant difference in the two sets of coefficients for each feature; in general, coefficients for the within condition are greater than the between condition. This is also true for the feature Loudness, which had only five X-head pairs with negatively correlated distributions (producing 10 pairs of coefficients). In other words, even with a relatively low *n*, there is a statistically closer relationship between the features of an X-head's main genre and their other music than with the features of a different X-head's other music, i.e., a significant influence of the main genre on music of secondary importance within people's downloads.

Despite the results having a clear direction, the current method was not able to test the influence of five of the 10 features within the analysis: Duration, Instrumentalness, Liveness, Speechiness, and Tempo. Although the observed pattern of influence may well extend to these features, this is by no means certain—for cognitive and neurological reasons, this phenomenon may be limited to particular acoustic features; for example, perhaps those that are more closely tied in some way to personality (e.g., McCown et al., 1997). Moreover, Analysis 1 was not able to address whether some X-head subgroups exhibited more influence, or if specific genres were more susceptible to being influenced by other genres more dominant within people's download collections. For example, is it the case that Classical is more prone to the influence of negatively valenced or sad music than, say, Metal? Similarly, might Jazz be more immune to the influence of up-tempo music than Bollywood, and what might be the interaction of X-head subgroup on these processes? Our aim was not necessarily to explain such patterns, which may well involve a combination of personality and sociocultural factors, but rather to observe the degree to which they existed within Nokia DB. To this end, we undertook a second analysis in which detailed information relating to each X-head subgroup and our selected 10 genres was brought to light.

## 4. Analysis 2: feature-influence matrices

### 4.1. Method

Each feature-influence matrix, referred to as C, was calculated from two submatrices, A and B. A, a 10 (X-heads) × 10 (genres) submatrix, contained the average feature values of all songs within a genre downloaded by an X-head subgroup (for example, the average value of Valence for all Reggae tracks downloaded by Classical-heads). B, a 10 (genres) × 1 (averages) submatrix, contained the average feature value of each genre downloaded by all users, excluding those made by the main X-head subgroup (for example, the average Valence of Metal tracks downloaded by everyone except Metal-heads). C, the 10 (X-heads) × 10 (genres) feature-influence matrix, was calculated by subtracting the row values in A (subtrahends) from those in B (minuends), and converting the resulting differences into percentage changes from the averages in B. Formally, the above is given by:

(1) $$\begin{array}{r} F=\left\{\forall x_{g}\in F_{xg},x_{g}=\left\{\frac{F_{xg}-P_{g}}{P_{g}}*100\right\}\right\} \end{array}$$

Where:

*F* = Feature-influence matrix (Matrix C)
*x* = X-head subgroup
*F_xg_* = Average feature value for genre (*g*) in X-head (*x*) subgroup (Submatrix A)
*P*_*g*_ = Average feature value for genre (*g*) for entire population (Submatrix B)
*x_g_* = Average feature value for genre (*g*) listened to by X-head subgroup (*x*)

We illustrated this process with reference to Submatrices A and B, Matrix C (the feature-influence matrix), and feature Valence. For clarity, the calculation is simplified to include only three X-head subgroups and genres: Dance, Metal, and Pop.

#### 4.1.1. Submatrix A

Table 2 shows Matrix A: rows (*i*) represent X-head subgroups; columns (*j*) represent genres downloaded by each X-head subgroup. For example, average Classical, Dance, and Metal Valence values for Dance-heads (*i* = 2, *j* = (1, 2, 3)) are (2, 1) = 0.28, (2, 2) = 0.41, and (2, 3) = 0.37 respectively.

**Table 2:** Example of Submatrix A showing the average Valence of three genres downloaded by three X-head subgroups.

	**Classical**	**Dance**	**Metal**
**Classical-heads**	0.27	0.43	0.38
**Dance-heads**	0.28	0.41	0.37
**Metal-heads**	0.28	0.44	0.35

*Rows represent X-heads; columns represent genres*.

#### 4.1.2. Submatrix B

Table 3 shows Submatrix B: the columns are genres; the row is the average Valence of each genre, excluding members of that particular X-head subgroup. For example, the average Valence for Metal music downloaded by non-Metal-heads (1, 3) = 0.39.

**Table 3:** Example of Submatrix B for feature Valence with three genres.

	**Classical**	**Dance**	**Metal**
Population average	0.28	0.47	0.39

*Average feature values of genres, excluding members of each particular X-head subgroup*.

#### 4.1.3. Matrix C (feature-influence matrix)

Table 4 shows Matrix C, generated by subtracting cell *i, j* in Submatrix A from cell *i, j* in Submatrix B. We take the percentage change for that feature using the population average for a particular genre in Submatrix B (similar results were obtained using population medians as opposed to averages). For example, to calculate cell (2, 2) of Matrix C:

$$\begin{array}{lll} C_{i,j} & = & \left(\frac{A\left(i,j\right)-B\left(j\right)}{B\left(j\right)}\right)*100\\ C_{2,2} & = & \left(\frac{0.41-0.47}{0.47}\right)*100=-12.8 \end{array}$$

This example indicates that Dance-heads downloaded Dance music that was 12.8% more negatively valenced than the rest of the population downloading Dance.

**Table 4:** Example of Matrix C, the feature-influence matrix, showing the percentage Valence change of three genres downloaded by three X-head subgroups.

	**Classical (%)**	**Dance (%)**	**Metal (%)**
**Classical-heads**	−3.57	−8.51	−2.56
**Dance-heads**	0.0	−12.8	−5.12
**Metal-heads**	0.0	−6.1	−10.26

*Rows represent X-heads; columns represent genres*.

### 4.2. Results

**Figure 6:**
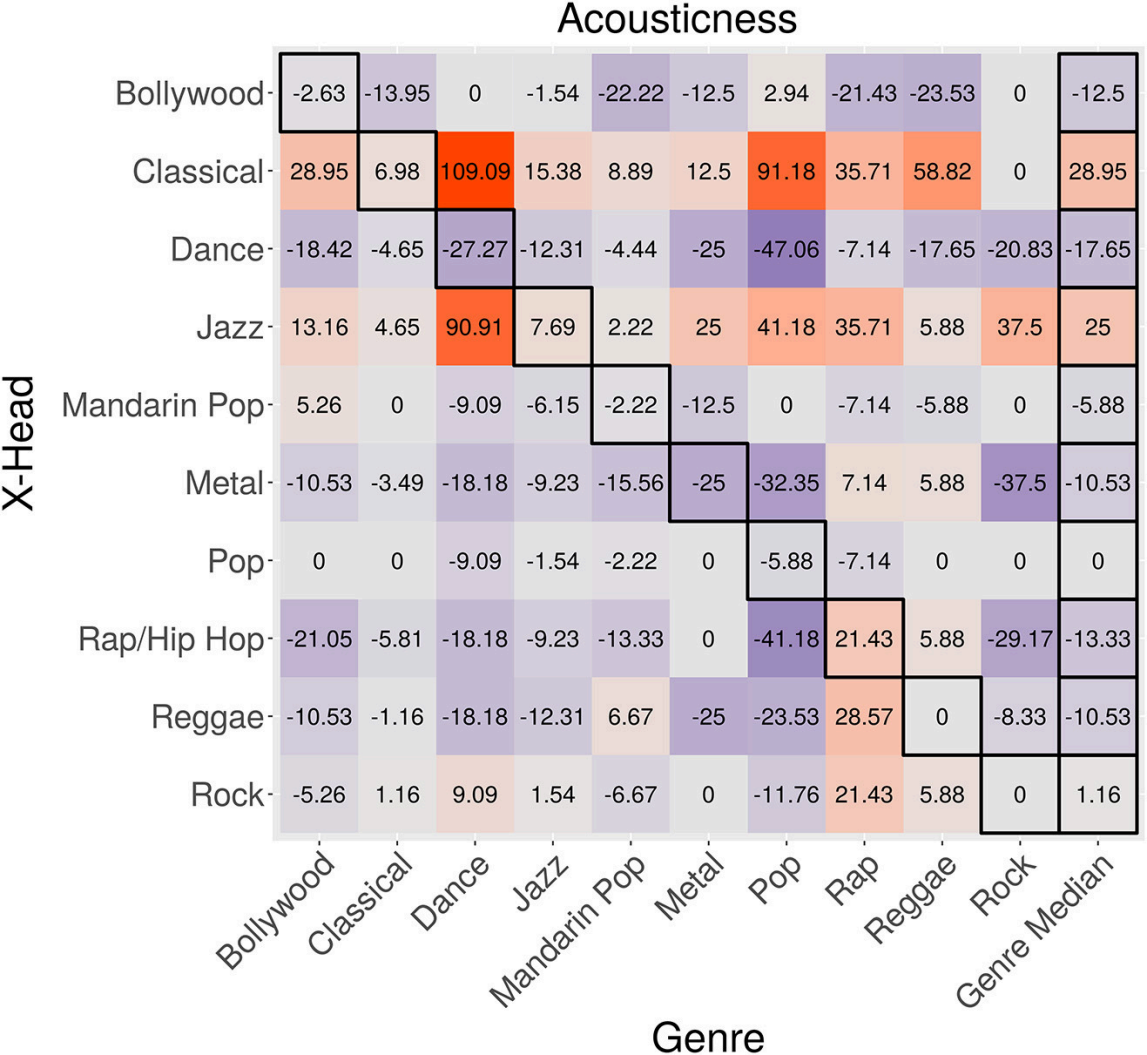
Feature-influence matrix for Acousticness. The highlighted diagonal cells (running top-left to bottom right) show X-heads with respect to their main genres. The highlighted column on the right shows the median value of each row, excluding diagonally highlighted cells.

Figure 6 shows the feature-influence matrix for Acousticness. Cell values indicate the percentage change in Acousticness of genres (columns) downloaded by X-head subgroups (rows), compared to the average Acousticness of genres downloaded by the overall population. The highlighted diagonal cells (running top left to bottom right) show X-heads with respect to their main genres. The highlighted column on the right shows the median value of each row, excluding diagonally highlighted cells.

**Figure 7:**
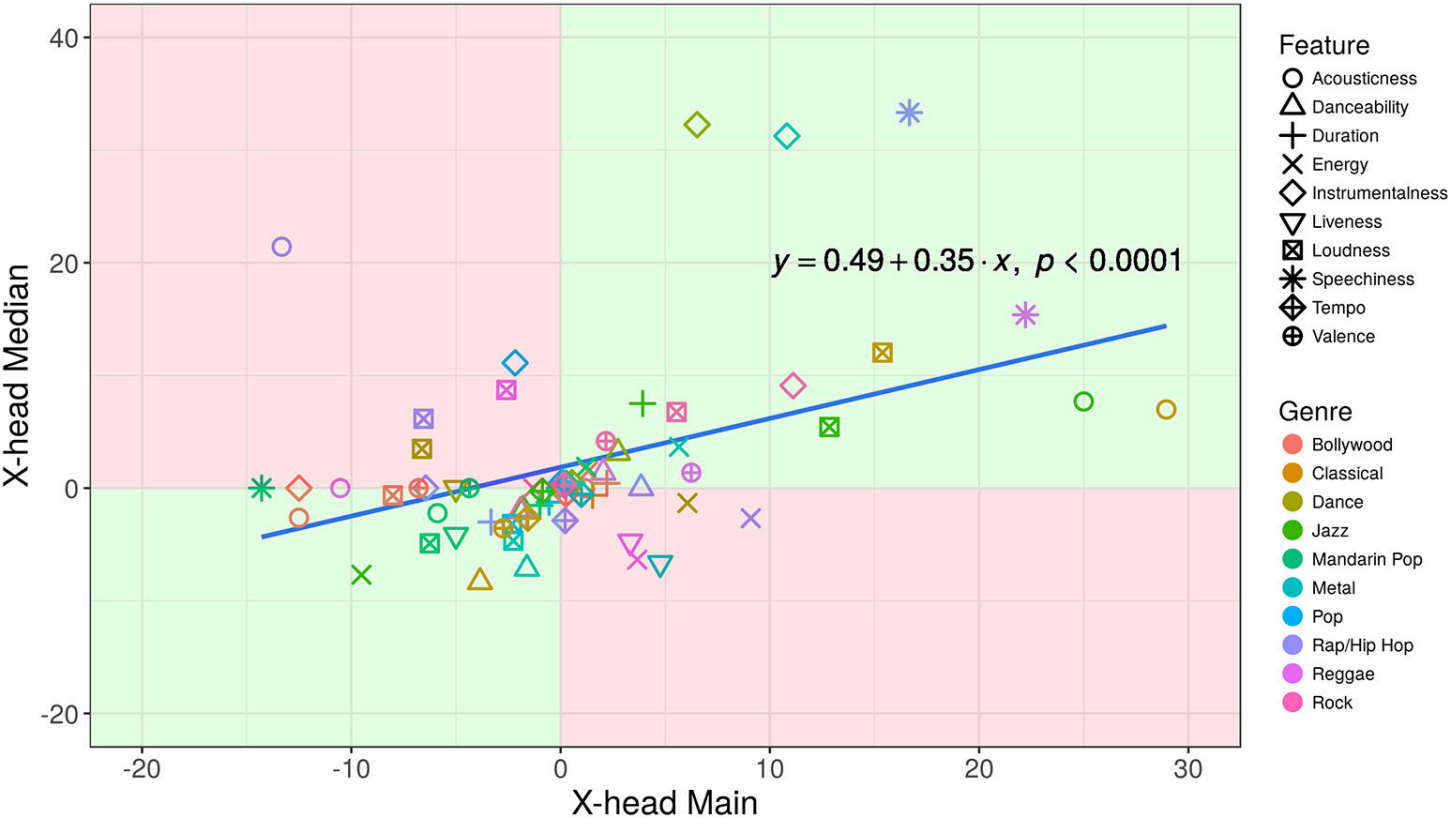
Scatterplot showing the 100 diagonal-to-median cell pairings of the 10 feature-influence matrices. Light-green quadrants indicate sign agreement between the row medians and X-heads with respect to their main genres, either positive or negative; pink quadrants indicate sign disagreement.

Of the 100 possible diagonal-to-median cell pairings (10 features × 10 X-heads), the signs of 64 were in agreement; 36 were in disagreement (see Supplementary Figure 2). These pairings are shown in the scatterplot in Figure 7. Light-green quadrants indicate sign agreement between the row medians and X-heads with respect to their main genres, either positive or negative; pink quadrants indicate sign disagreement. The adjusted *r*^2^-value, 0.1039, gives rise to the following statistic: *r* = 0.34, *n* = 100, *p* < 0.0001.

The 10 feature-influence matrices enabled two further, complementary questions to be explored. First, across all X-heads, which feature of their main genres most strongly influenced their other genres? For example, is the relationship between X-heads' main and other genres stronger for Energy than Danceability? This question was assessed by correlating X-heads' main genres with the nine other genres in each of their download collections. This produced one overall coefficient per feature-influence matrix; the resulting 10 coefficients were then ranked in order. The second question asked which X-head subgroup across all features had the closest relationship between their main genre and other genres. For example, do the features of Mandarin Pop-heads' main genre more strongly influence the corresponding features of their other genres than is the case for Reggae-heads? This question was investigated by correlating each X-head's main genre with the nine other genres in each feature-influence matrix. This produced one overall coefficient per X-head subgroup, and, as before, the resulting 10 coefficients were ranked in order. The results of these analyses are shown in Tables 5, 6. Rows represent ranks, either of features or X-heads. Also shown are associated Pearson product-moment correlation coefficients.

**Table 5:** Table showing the ranked order of features.

**Rank**	**Feature (*n* = 90)**
1	Speechiness; *r* = 0.52^***^
2	Danceability; *r* = 0.48^***^
3	Loudness; *r* = 0.45^***^
4	Energy; *r* = 0.44^***^
5	Acousticness; *r* = 0.28^**^
6	Tempo; *r* = 0.19
7	Duration; *r* = 0.17
8	Valence; *r* = 0.14
9	Liveness; *r* = 0.06
10	Instrumentalness; *r* = 0.04

The feature column shows which feature of X-heads' main genres is closest to their other genres (p < 0.1; ^*^p < 0.05;

** p < 0.01;

*** *p < 0.001)*.

**Table 6:** Table showing the ranked order of X-heads.

**Rank**	**X-head (*n* = 90)**
1	Metal; *r* = 0.56^***^
2	Jazz; *r* = 0.49^***^
3	Dance; *r* = 0.45^***^
4	Classical; *r* = 0.41^***^
5	Rap; *r* = 0.28^**^
6	Rock; *r* = 0.26^*^
7	Bollywood; *r* = 0.14
8	Reggae; *r* = 0.11
9	Mandarin Pop; *r* = 0.09
10	Pop; *r* = 0.08

The X-head column shows the subgroup with the closest relationship between their main genre and other genres, across all features (p < 0.1;

* p < 0.05;

** p < 0.01;

*** *p < 0.001)*.

### 4.3. Discussion

The finding in the feature-influence matrices that the signs of 64 diagonal-to-median cell pairings were in agreement, with 36 in disagreement, strongly suggests that there is a directional relationship, either positive or negative, between the features of X-heads' main genres and those of their other genres. This is confirmed in the scatterplot in Figure 7, and associated correlation statistic (*r* = 0.34), in which there was a significant, positive relationship between the variables. Of course, our assumption is that the direction of influence is from the main to the other genres in each X-head subgroup: intuitively, at least, it seems to make sense that most individuals have a preferred musical style that influences their choices in other genres. However, the converse could be true: the features of X-heads' secondary genres influence the choices they make in their main genre, although this is perhaps less likely.

In the foregoing diagonal-to-median cell analysis, two additional analyses sought to establish ranked orders showing: (1) which feature of X-heads' main genres most strongly influenced their other genres, and (2) which X-head subgroup's main genre most strongly influenced their other genres across all features. In Table 5, the top-ranked feature was Speechiness (*r* = 0.52)—the presence or absence of spoken words in tracks belonging to main genres appears to have created a preference for similarly “speechy” tracks in X-heads' other genres. Similarly, the Danceability, Loudness, and Energy of users' predominant tracks appear to have heavily influenced tracks of secondary importance. At the other end of the spectrum, there was little-to-no relationship between X-heads' main and other genres in terms of Liveness (whether a track was recorded at a live event) and Instrumentalness (whether a track was created using only instrumental sounds).

The top-ranked X-head subgroups were Metal (*r* = 0.56) and Jazz (*r* = 0.49). The dynamic nature of much Metal music seems to have created a musical ‘fingerprint’ that strongly expressed itself in the other genres Metal-heads downloaded. Likewise, Jazz-heads seem compelled to seek out music containing Jazz-like qualities when exploring non-Jazz music. Conversely, Mandarin Pop and Pop's musical features did not significantly influence the other music downloaded by these X-head subgroups, perhaps because the features of these genre are relatively indistinct (*r* = 0.09 and *r* = 0.08 respectively). These findings, and those relating to Analysis 1, are now discussed in the broader context of the paper.

## 5. General discussion

Using pairs of X-head subgroups whose feature distributions negatively correlated, Analysis 1 found that there was a consistent relationship between X-heads' main and other genres; the methodology enabled five acoustic features to be investigated. Analysis 2 added detail to this finding through the production of 10 feature-influence matrices; there was a significant positive correlation between the diagonal-to-median cell pairings across the matrices, again strongly indicating that there is a relationship between the features of X-heads' main genres and those of their other genres. Therefore, with respect to the question posed at the outset, the core findings of this paper support the proposition that the acoustic features of a person's main musical genre influence their choices within non-preferred, secondary styles. Which is to say, attributes of the tracks within preferred genres influence the other genres of people's music-download collections. The nature of this influence, and its possible actuating mechanisms, form the major part of the following discussion.

Although, as outlined in Section 1, substantial research has been undertaken in relation to musical preference and personality, for a variety of reasons relatively few studies have explored this issue using large music-consumption databases, such as Nokia DB. First, in terms of usual research timescales (i.e., years, not months), APIs, through which large volumes of data become accessible to external researchers, are relative newcomers to the academic landscape. Second, API rate-limits typically restrict the amount of data that is available; similarly, database limits may constrain the type of information that a researcher is able to export. And third, for sound methodological reasons relating to data integrity, psychologists have tended to rely on relatively small subject pools to whom individual personality or self-image tests can be administered (e.g., Zweigenhaft, 2008; Krause and Hargreaves, 2013).

In seeking to corroborate the findings of preexisting music-personality studies, Bansal and Woolhouse (2015), using Nokia DB, investigated (1) whether X-head subgroups showed distinct patterns of genre exclusivity, and, if so, (2) whether genre exclusivity related to the Big Five personality factors (Costa and MacCrae, 1992). X-heads ranked from exclusive to inclusive were as follows: Pop, Dance, Rap, Metal, Rock, Classical, Country, Folk, Jazz, and Indie. Interestingly, this aligned with previous literature showing that individuals who prefer Jazz and Folk score highly in the Big Five factor of openness (Zweigenhaft, 2008). Those high in openness were also found to avoid genres like Pop; likewise, Bansal and Woolhouse (2015) determined Pop-heads to be the most genre exclusive. In sum, genre-openness (and –agreeableness) associations from Zweigenhaft (2008) predicted genre inclusivity in Nokia DB X-heads—individuals with high openness scores (and/or agreeableness) were more likely to have a wider selection of genres within their music collections. Bansal and Woolhouse (2015) did not find conscientiousness, extraversion, or neuroticism to be predictors of genre exclusivity.

By demonstrating that personality-related behavior is discernable within big data concerning music consumption, Bansal and Woolhouse's (2015) research is highly relevant to the current study. If personality can be shown to have guided genre exclusiveness, then its involvement in other aspects of people's musical choices is not only possible, but, arguably, probable. In the present instance, a mechanism is being sought that may account for influence in terms of acoustic features and genres of secondary importance within X-heads' downloads. McCown et al. (1997) linked exaggerated bass frequencies, i.e., a specific acoustic feature, to a particular personality factor, neuroticism—it would seem self-evident that other acoustic features, including those explored within our study, will likewise be linked to aspects of personality, and therefore expressed *throughout* individuals' music collections. For example, in Table 6 Dance-heads are the third most influence-exhibiting subgroup (*r* = 0.45), indicating that the feature values of their main genre were significantly related to those of their other genres. Similarly, Danceability, a feature strongly associated with Dance-heads (see Figure 1), also ranks highly in Table 5 (*r* = 0.48). Given the work of McCown et al. (1997) linking Dance with neuroticism, it is tempting to conjecture that the increased feature influence of Dance-heads and Danceability is in someway related to heightened obsessiveness, a trait strongly associated with neurotic tendencies (Samuels et al., 2000). However, although intriguing, this proposition is beyond the scope of the present study, and thus awaits further investigation.

Alongside our fledgling personality hypothesis, expounded above, the work of Berns et al. (2010) and Halko and Kaustia (2015), discussed briefly in Section 1, is suggestive of neurophysiological mechanisms underpinning musical-feature influence. Specifically, we address this issue with reference to Aniruddh Patel's research involving music, language, and statistical learning—the ability of humans and other animals to acquire implicit knowledge about the world through the extraction of statistical regularities within their environments (Friedman et al., 2001; for neurological evidence concerning statistical learning of language, see Cheour et al., 1998; Rivera-Gaxiola et al., 2005). In order to account for the finding that the prosodies of English and French are reflected in the rhythms and melodies of these countries' respective instrumental music, Patel proposes a “direct-route” hypothesis, in which “statistical learning of the prosodic patterns of speech creates implicit knowledge of rhythmic and melodic patterns in language, which can in turn influence the creation of rhythmic and tonal patterns in music” (Patel et al., 2006, p. 3043). In other words, statistically acquired sound-pattern knowledge “leaks” from the domain of language, resulting in the rhythmic and melodic modification of music. Typically assessed using the Normalized Pairwise Variability Index (nPVI), a technique that measures the degree of durational contrast between successive elements in a sequence, research demonstrating this phenomenon is both robust and compelling (e.g., Huron and Ollen, 2003; Patel and Daniele, 2003; Daniele and Patel, 2004).

Patel's work is highlighted here by way of analogy—the phenomenon of musical-feature influence is limited to music, and therefore is not a cross-domain effect. However, statistical learning may well be pertinent to our findings, and suggests the existence of a mechanism that is more or less independent of personality (to our knowledge, no research has linked personality factors with abilities in statistical learning). Given empirical evidence of temporal and intervallic relationships between music and language, and Patel's assertion that this is underpinned by statistical learning and hence causal in nature, it is plausible to suggest that a similar process operates with respect to musical features. That is, listeners extract the statistical regularities of musical features, which in turn influence the creation of musical preferences beyond established style boundaries and/or genre categories. Statistical regularities of features may be relatively straightforward, such as Tempo—the speed of the most salient pulse in the music, usually measured by allowing listeners to tap along to perceptually noticeable beats (McKinney and Moelants, 2006)—or complex, such as Danceability—an amalgamation of tempo, rhythm stability, beat strength, and isochrony.

In Table 5, the effect of Tempo on secondary genres was only marginally significant, whereas Danceability was highly significant. If statistical learning is at play, this finding suggests that its effect is bolstered through the presence of multiple, mutually reinforcing acoustic components, as is the case for Danceability. Arguably less plausible, however, is the notion that statistical learning affects X-head subgroups differentially. If this were the case, the rankings in Table 6 would indicate that Metal-heads, who are at the top of the table, engage in statistical learning, whereas Pop-heads, at the bottom, do not. While this seems unlikely, it might be that Metal is acoustically more regular than Pop, and therefore facilitates statistical learning to a greater degree; although, given the high level of signal redundancy in much Pop music, this hypothesis would seem to be doubtful.

### 5.1. Limitations

In presenting our findings we have attempted to develop and adapt a range of approaches, suitable to the data at hand. And while the premise of the question motivating our research is supported by a series of cogent results, the adopted methodologies, as well as the data themselves, are limited in a variety of ways and raise a number of questions.

First, the algorithms responsible for Spotify's acoustic features are proprietary, and therefore not publically available. As a result, although our primary aim was to investigate and record the presence of musical-feature influence, we were unable to assess in detail which specific acoustic elements were responsible for our findings. Which frequency bands within an X-head's main genre, for example, have in general a greater influence on their other genres? Which components of Acousticness are present throughout an X-head's download collection, and which are specific to their main genre? Moreover, and perhaps of greater import, as mentioned at the outset, the psychological reality of acoustic features is, as yet, unquantified (Friberg and Schoonderwaldt, 2014). Although a feature like Valence may make sense to those who know and love music's emotional power, its interpretation across listeners may be highly divergent. Valence is frequently characterized with reference to mode, either major (positive/happy) or minor (negative/sad) (Kastner and Crowder, 1990). However, those familiar with works such as Elgar's “Nimrod” (*Enigma Variations, Op. 36*), which although in a major key is deeply poignant, may take a very different view of this dichotomy.

Second, no attempt has been made to address the issue of mood, referred to in Section 1. As discussed, in contrast to the stability of personality, mood is thought to change relatively rapidly (McFarlane et al., 1988). Our analyses did not take into account temporal order or download timelines, which may have revealed day-to-day effects of mood. For example, an important question might be, do downloads oscillate between negative and positive Valence, and, if so, is the influence of the upswing to positive different from the downswing? Although this question is beyond the scope of the present study, and would no doubt require very different methodologies to those used here (e.g., time-series analysis), the Nokia DB does contain detailed date/time information that would, in theory, enable this matter to be addressed.

Third, as mentioned in Section 2, our intention was to define X-heads straightforwardly, i.e., a majority of downloads in a particular genre. While this simple metric has the advantage of transparency—X-heads are not cooked up using a complicated, opaque recipe—the approach will undoubtedly have created a class of users with overlapping, ill-defined boundaries, which could have introduced undue noise into the analyses. In this respect, no attempt was made to separate “Super-heads,” e.g., users in the upper quartile in terms of main-genre proportion, from “Weak-heads,” e.g., users in the lower quartile. And, consequently, some users in different X-head subgroups may have been similar. For example, consider two users, P and Q, with the following download proportions: P = 55% Jazz, 45% Classical; Q = 55% Classical, 45% Jazz. Despite P and Q having a great deal in common, our method would group them as categorically distinct: P a Jazz-head, Q a Classical-head. The question then arises as to whether feature influence is more accentuated in Super-heads vs. Weak-heads (which we would imagine to be the case), or whether no such effect exists. While the downside of our simple X-head definition was that this issue could not be addressed, the upside is that the data within Nokia DB, with a little preprocessing, affords us the opportunity to answer this question in detail in the future.

### 5.2. Closing remarks

In summary, Analyses 1 and 2 found strong evidence of influence with respect to users' consumption of multiple styles of music; clear relationships emerged between the features of X-heads' main and secondary genres. This effect was found to be stronger for some features than others, most noticeably Speechiness, Danceability, and Loudness, and more pronounced in certain subgroups, such as Metal-heads, Jazz-heads, and Dance-heads. While the reasons for differential effects within features and X-heads is unknown, two probable, independent causal mechanisms were suggested to account for main-to-secondary genre influence. First, personality creates an overarching psychological framework in which certain factors, such as openness and agreeableness, guide musical preference, irrespective of genre; some personality factors may be linked to specific acoustic features. Second, via statistical learning, listeners extract the acoustic regularities of various musical features, which in turn influence the creation of musical preferences beyond favored styles and/or genres. Of course, these mechanisms need not be mutually exclusive, but may serve to reinforce one another. Attempts, therefore, to tease apart the effects of personality and statistical learning could prove to be difficult, although paradigms in which these factors are independently manipulated might settle the issue of personality vs. statistical learning conclusively.

## Ethics statement

This study was carried out in accordance with the recommendations of the Research Ethics Board of McMaster University, Canada. The protocol was approved by the McMaster Research Ethics Committee.

## Author contributions

MB: Study design and execution, data analysis and interpretation, figure and graph creation, and manuscript review. JB: Study design and manuscript review. MW: Manuscript drafting, study design, data analysis and interpretation.

### Conflict of interest statement

The authors declare that the research was conducted in the absence of any commercial or financial relationships that could be construed as a potential conflict of interest.

## Footnotes

^1^Nokia DB represents a portion of Nokia's total commercial activity, and is therefore not indicative of market share.

^2^https://developer.spotify.com/web-api/

^3^Online information can be accessed at the following webpage: https://web.archive.org/web/20150112031805/http://developer.echonest.com/acoustic-attributes.html

## Supplementary material

Supplementary Figure 1 Correlation matrices for all acoustic features (Acousticness, Danceability, Duration, Energy, Instrumentalness, Liveness, Loudness, Speechiness, Tempo, Valence), as described in Section 3.1, Figure 3.

Supplementary Figure 2 Feature-influence matrices for all acoustic features (Acousticness, Danceability, Duration, Energy, Instrumentalness, Liveness, Loudness, Speechiness, Tempo, Valence), as described in Section 4.1, Figure 6.
