# Supplementary figures and images for: Acoustic Features Influence Musical Choices Across Multiple Genres
Source: Front Psychol. 2017 Jul 4;8:931. doi: 10.3389/fpsyg.2017.00931 (PMC5495864; doi:10.3389/fpsyg.2017.00931)

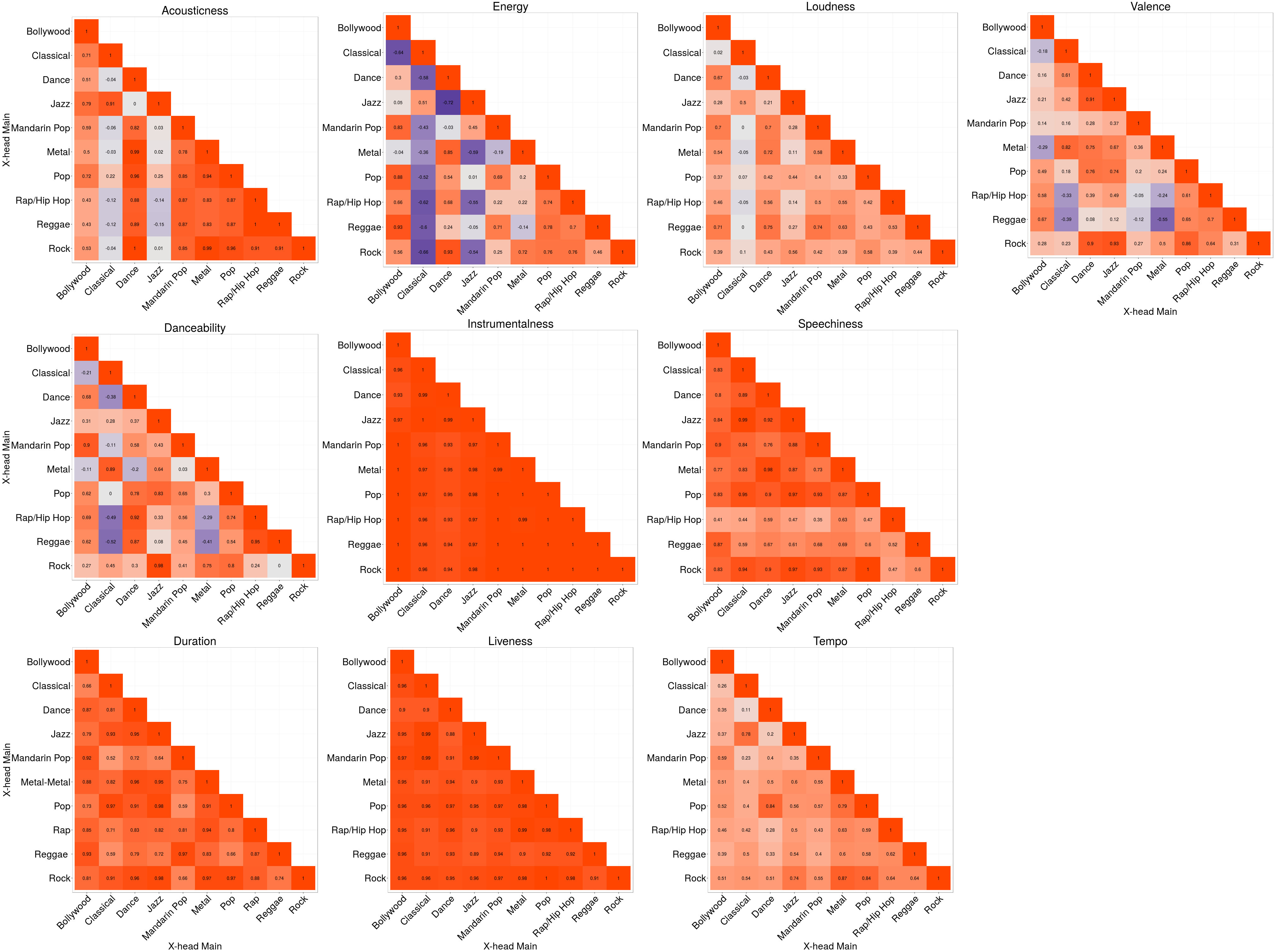

Supplement: Supplementary Figure 1 — Correlation matrices for all acoustic features (Acousticness, Danceability, Duration, Energy, Instrumentalness, Liveness, Loudness, Speechiness, Tempo, Valence), as described in Section 3.1, Figure 3. [file Image1.JPEG]

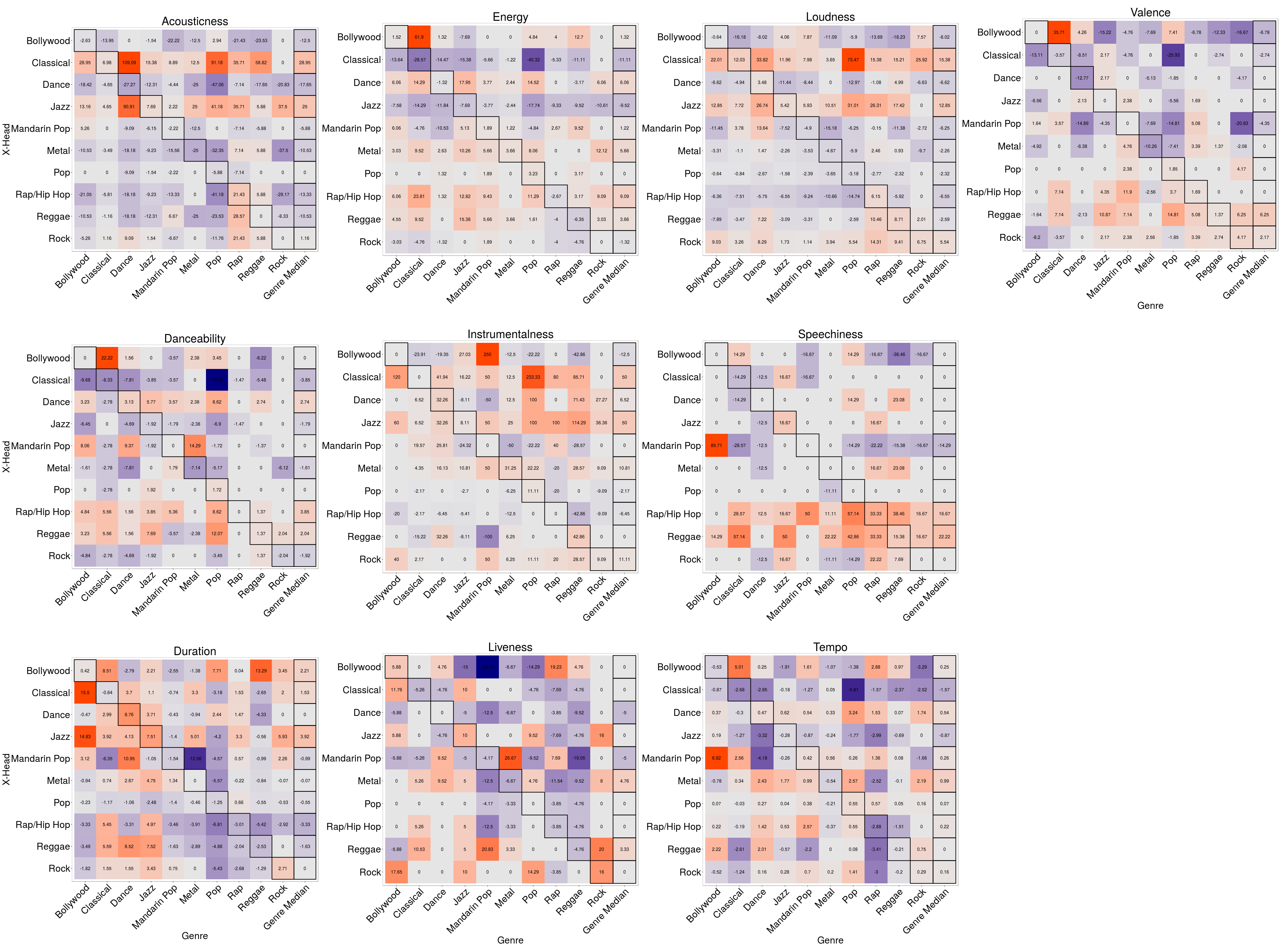

Supplement: Supplementary Figure 2 — Feature-influence matrices for all acoustic features (Acousticness, Danceability, Duration, Energy, Instrumentalness, Liveness, Loudness, Speechiness, Tempo, Valence), as described in Section 4.1, Figure 6. [file Image2.JPEG]
